# Employability and Job Insecurity: The Role of Personal Resources on Work-Related Stress

**DOI:** 10.5964/ejop.1904

**Published:** 2021-05-31

**Authors:** Tiziana Ramaci, Palmira Faraci, Giuseppe Santisi, Giusy Danila Valenti

**Affiliations:** a Faculty of Human and Social Sciences, University of Enna “Kore”, Enna, Italy; b Department of Educational Sciences, University of Catania, Catania, Italy; University of Wollongong, Wollongong, Australia

**Keywords:** employability, psycho-social distress, personal resources, organizations

## Abstract

This study is aimed to assess the effect of both employability and personal resources, in terms of pro-activity and self-efficacy, on the relationship between job insecurity and psycho-social distress. Using survey data from 211 participants, among employed, unemployed and workers in transition, we analyzed the incidence of employability, pro-activity and self-efficacy on psycho-social distress. Our results showed that the above-mentioned variables significantly differed by participants’ gender and age. The structural theoretical model proposed to assess the significance of the hypothesized paths exhibited good fit with the data. Thus, all our hypotheses were supported. Findings are in line with previous research, and practical implications may give significant effects when applied in new labor policies undertaken by local governments.

Nowadays’ sociopolitical and economic conditions seem more and more often characterized by multifarious and non-uniform connotations, more discontinuous than in the past. Work is not only a place where one’s identity can be set up or just one of the major social integration factors. It is also a source of personal wellness. Consequently, it is needed that working conditions fulfill at least some basic requirements: a job has to be reasonably stable, well paid, interesting enough, safety and dignity are totally respected. In other words, a job should comply with requirements for *dignity* and *meaning,* for both employers and job seekers (Blustein, 2011; Massimiani, 2007, 2010) respecting the characteristics of *quality*.

Taking into account the European Committee’s official guidelines (European Commission [2001]), the idea of *job quality* is referred to a complex and multi-dimensional concept, since it considers not only the opportunities of paid jobs, but also their characteristics. According to Massimiani (2010), these properties correspond to: 1) the objective characteristics of the workplace; 2) the worker’s specific characteristics; 3) the harmony between a worker’s specific characteristics and the requirements for the activity to be performed; 4) the level of personal satisfaction.

In order to have a unitary analysis framework, the same European Commission (2001) identified a number of *job quality* components, included in two macro-categories: 1) the characteristics of the workplace, regarding the intrinsic quality of the job (type of contract, work shifts, competence required), and life-long learning and career development processes; 2) the characteristics of workplace and labor market (gender equality, work health and safety, flexibility and inclusion, work-life balance, diversity and productivity).

The aim of the present study was to analyze the relationship between job insecurity and psycho-social disturbances during job transitions or when people start working after a jobless period. Specifically, the main goal was to verify how two personal resources—self-efficacy and pro-activity—together with one’s own perception of employability may mediate the relationship between one’s status of insecurity and situations of unease or illness.

## Job Flexibility, Job Insecurity and Personal Resources

The concept of "precariousness" that we use is that which emerges clearly from the meaning attributed to it in some "economically advanced" countries (Standing, 2011). Regardless of the possible and different interpretations, the perception of job insecurity undoubtedly represents a clear risk factor for the health of individuals and their families, ending up as one of the main sources of different forms of psychological distress and discomfort (Girardi et al., 2015). The extreme discretion of most jobs in the modern European market and the consequent flexibility of work are certainly the main reasons for job insecurity. Autonomy, independence and new technologies have brought us towards a work individualization process and an increased sense of insecurity due to the consequent occurrence of profiles bound to have “boundaryless” careers; this gain even more importance in the case of atypical workers. The status of “job insecurity” lies in an extremely flexible context, between having a job activity and being jobless. Its perception varies between the “subjective” and the “objective,” depending on the real risk of losing one’s job. The impact of this condition on health is remarkable and several authors argue that it can be more powerful than joblessness. The recessionary cycles that can periodically affect more or less developed geographic areas, or even global health crises such as the recent coronavirus disease (COVID) pandemic, cause unexpected shocks on the employment systems to the point of requiring absolutely new and exceptional occupational recovery strategies, totally distant from the application of traditional tools for effective employability of workers (Buffel, Velde, & Bracke, 2015; Giorgi, Arcangeli, Mucci, & Cupelli, 2015; Mucci, Giorgi, Roncaioli, Fiz Perez, & Arcangeli, 2016). Precisely in relation to the COVID crisis, based on preliminary estimates by the International Labor Organization (ILO), the economic and labor crisis caused by COVID-19 could increase unemployment in the world by almost 25 million. The estimates indicate an increase in global unemployment from 5.3 to 24.7 million. This would add up to 188 million unemployed people in the world in 2019. As the ILO estimates that between 8.8 and 35 million more people will find themselves in conditions of working poverty worldwide, the same organization has outlined guidelines guides that aim to provide immediate responses to the "unemployment" emergency. Of all, we limit ourselves to: a) strengthening of measures on health and safety at work; b) restructuring of the organization of work with smart working tools; c) wider access to health for all workers; d) strengthening the capacity and resilience of employers' and trade union organizations; e) strengthening of social dialogue, collective bargaining and industrial relations; f) strengthening of the governance (ILO, 2020; Green, 2020; Kazmi, Hasan, Talib, & Sagar, 2020).

Regarding the above-mentioned aspects, most recent studies on employability (Cai et al., 2015; Kim, Kim, & Lee, 2015; Lodi, Zammitti, Magnano, Patrizi, & Santisi, 2020; Lo Presti, Pluviano, & Briscoe, 2018) have shown that the most important set of personal resources needed to manage one’s unease deriving from job insecurity are mostly to be found in everyone’s Psychological Capital (PsyCap). This is both in its original structure and formulation (Luthans, Youssef, & Avolio, 2007), and in the more recent attempts to reinforce the idea through new constructs, like Emotional Intelligence (Di Fabio & Palazzeschi, 2016; Magnano, Santisi, & Platania, 2017), Risk Intelligence and Courage (Magnano, Paolillo, Platania, & Santisi, 2017).

PsyCap can be described as the psychological state of an individual who is able to: 1) focus on his or her self-efficacy in order to be able to perform demanding tasks; 2) carry out positive assignments—optimism—on what is happening now and what could happen in the future; 3) persevere towards one's goals and, if necessary, redirect the paths towards the desired goals—hope—to achieve success; 4) get together and go further to achieve success—resilience—if affected by problems and adversity (Luthans et al., 2007).

Although each of the four components of PsyCap is conceptually and empirically independent, research has shown that it has a common psychological substrate that actually binds them together (Luthans & Youssef, 2007) and which represents its added value, the latent dimension that holds all secondary dimensions together. Individuals with higher levels of PsyCap have greater psychological resources and, in organizational contexts, enjoy greater advantages than those with high levels of one or two dimensions. Taken in its entirety, PsyCap offers a further contribution to predicting future performances with respect to its secondary dimensions considered separately (Consiglio & La Mura, 2015; Luthans & Youssef, 2007).

Moreover, PsyCap goes beyond the consolidated theories about the human capital (“what you know”), emphasizing the aspects of “who you are” and “what you are turning into” (Avolio & Luthans, 2006; Luthans & Avolio, 2004). Also, for this reason, the PsyCap multidimensional and dynamic construct is complemented by specific motivational configurations and behavior styles, expression of an individual’s subjective potentiality, that is a personal and valuable endowment linked to adaptive decisional strategies to overcome obstacles and achieve the goals set. For the purpose of this survey and its operative design the authors have used only one of the constructs constituting the Psychological Capital, that is the perception of self-efficacy.

In accordance with the theoretical pattern proposed by Luthans and Youssef (2004), the first PsyCap sub-dimension is self-efficacy, directly derived from Albert Bandura’s socio-cognitive theory (Bandura, 1986). It is described as the “belief of having the ability to organize and carry out action sequences required to produce certain results which have an impact on the subject’s commitment, the choice of the objectives and the performance” (Luthans & Youseff, 2004, p. 153).

Generally, self-effective people choose more difficult tasks and more stimulating objectives because they know they are able to succeed; their motivation increases in complex situations and their perseverance enhances in front of obstacles. Researchers have also showed that self-efficacy is accompanied by a greater spirit of initiative, higher levels of determination and motivation (Bandura, 1997; Bandura & Locke, 2003), higher job satisfaction and perceived wellness, lesser turnover and, obviously, better job performances (Avey, Luthans, & Jensen, 2009; Hodges, 2010; Luthans et al., 2007; Platania, Santisi, Magnano, & Ramaci, 2015).

## Employability

It is now clear that job insecurity conditions can be effectively (as a process) and positively (in terms of results) moderated and managed only by the workers’ objective skills, expressed according to their possibility to “re-employ themselves” in the current chaotic labor market. In other words, we need to try to understand which employability thresholds an individual can express to pursue the objective of a “dignified job” and properly face the psycho-physical impact of his/her job insecurity condition.

Some of the examined aspects are represented by alternatives to linear and hierarchical career patterns (Arthur & Rousseau, 1996; Pieperl & Baruch, 1997), as well as changing psychological contracts (Herriot & Pemberton, 1996; Rousseau, 1995, 2001; Sparrow, 1996). Most researchers focused on management matters such as the negative impact these trends may have on people’s attitude at work and on their affection towards their work company. Some authors (Baruch, 1998) argue that the traditional organization affection is to be wished for employees and a more individual work and career pattern is to be promoted, underlining the aspects which go beyond the organization, like the no-limit career (Arthur, 1994; Bird, 1994).

Changing from stable and long term into shorter, flexible and unsteady work relationships (Hiltrop, 1995), employable individuals generally seek places which can offer them career opportunities and possibilities of improving their know-how and skills (Baruch, 2001; Kluytmans & Ott, 1999). Some authors (Berntson, Näswall, & Sverke, 2010) believe that those who think of themselves as “employable” perceive themselves as if they had more opportunities in the labor market, and they will likely depart from the organization more rapidly than those who “do not feel employable.”

Due to its contradictory and confused nature, the idea of employability has often been doubted upon. Among the various critical approaches, though all dating back to the last two decades (Pascale, 1995; Rajan, van Eupen, Chappe, & Lane, 2000) Sanders and de Grip’s (Sanders & de Grip, 2004) can probably be considered as the most realistic, especially because they maintain that the meaning of employability has changed over the last 30 years, depending on the labor market conditions and policies of the time at play.

Rothwell and Arnold (2007) consider employability as a psycho-social construct (Fugate, Kinicki, & Ashforth, 2004) and define it as the ability to maintain the current job or to get the wished one; as such, it comprises: 1) *individual characteristics*, like knowledge and skills (Hillage & Pollard, 1998), learning abilities (Bagshaw, 1996; Lane, Puri, Cleverly, Wylie, & Rajan, 2000), mastering in managing one’s own career and in job searching (Hillage & Pollard, 1998), resilience (Iles, 1997; Rajan, 1997; Rajan et al., 2000), and self-efficacy; 2) *intra-organizational factors,* regarding the present and future conditions of domestic labor markets; 3) *external factors,* such as the foreign labor market conditions (Kirschenbaum & Mano-Negrin, 1999; Lane et al., 2000), which include those factors linked to job demands (Mallough & Kleiner, 2001).

**Figure 1:**
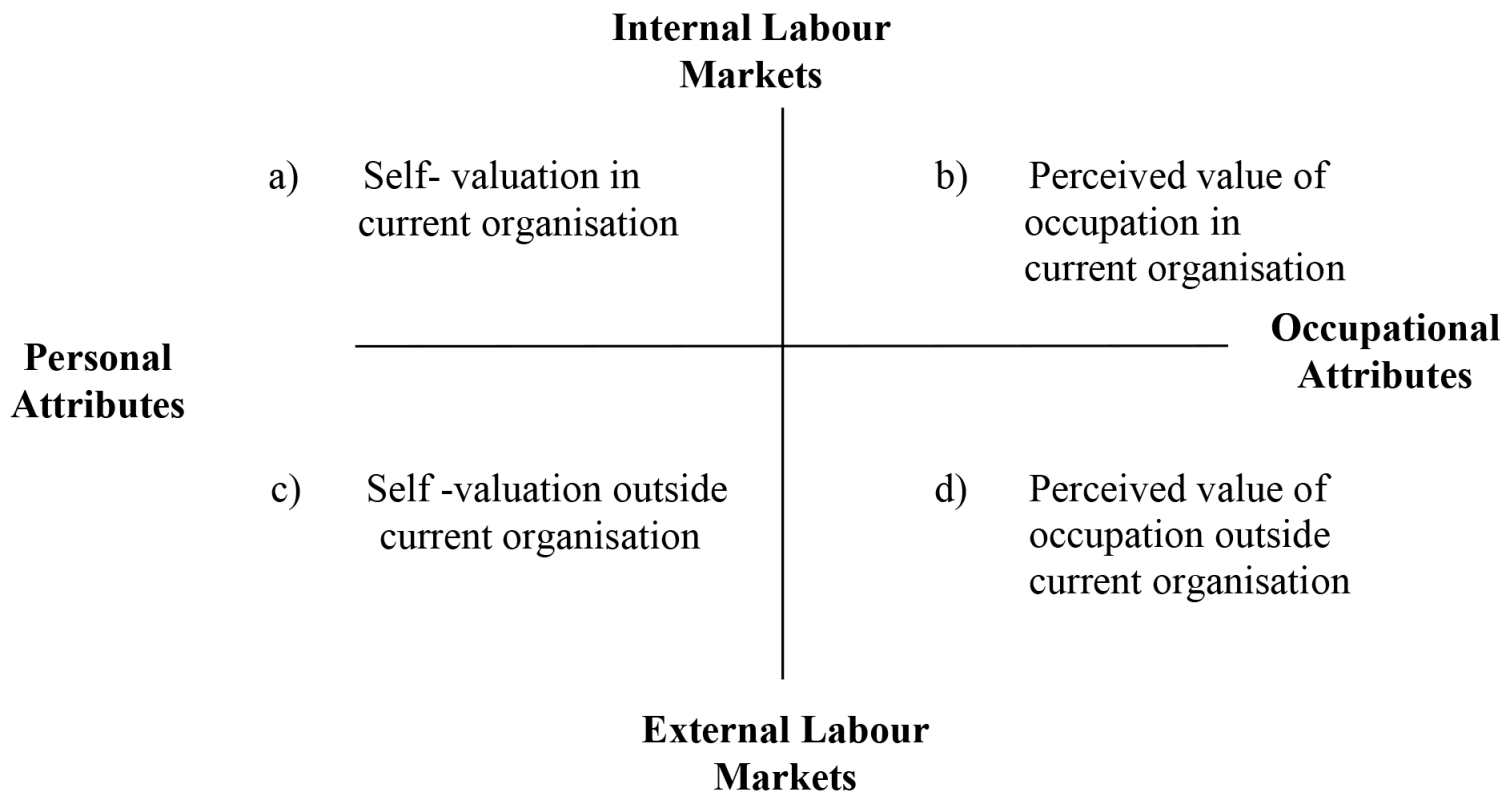
Graphic Chart of Employability Conditions (Rothwell & Arnold, 2007)

In their pattern, Rothwell and Arnold (2007) systematically show the major dimensions’ employability derives from, identifying two hypothetical lines whose ends are the foreign and domestic labor markets and the individual and work resources. The combination of these two dimensions originates four frameworks expressing the subject’s employability conditions (cfr. Figure 1): frame “*a*” represents the individuals’ evaluation of their own usefulness inside their work organization; frame “*b*” reflects the way the organization appreciates its manpower or professional team in the domestic labor market; frame “*c*” relates to the individual self-perception of one’s own job (mainly based on their personal competencies compared to their job characteristics) in the foreign labor market; frame “*d*”, finally, refers to how the foreign labor market is assessed and perceived by those with the individual’s personal experiences. These changes from job to job can also change along the way (Magnano, Santisi, Zammitti, Zarbo, & Di Nuovo, 2019).

## Aims and Hypotheses

On the basis of the foregoing premises, job security and employability thus appear as the two constructs better able to interpret the tax flexibility requirements from the labor market. It is necessary to associate a series of potentially suitable individual resources with these two concepts for predicting the possible health consequences of a job flexibility condition.

The study research design presented below investigates the relationship between job insecurity and psychological distress. Specifically, we aim to analyze the relationship between job insecurity, employability, two individual resources (i.e., pro-activity and self-efficacy) and psychological distress.

**Figure 2:**
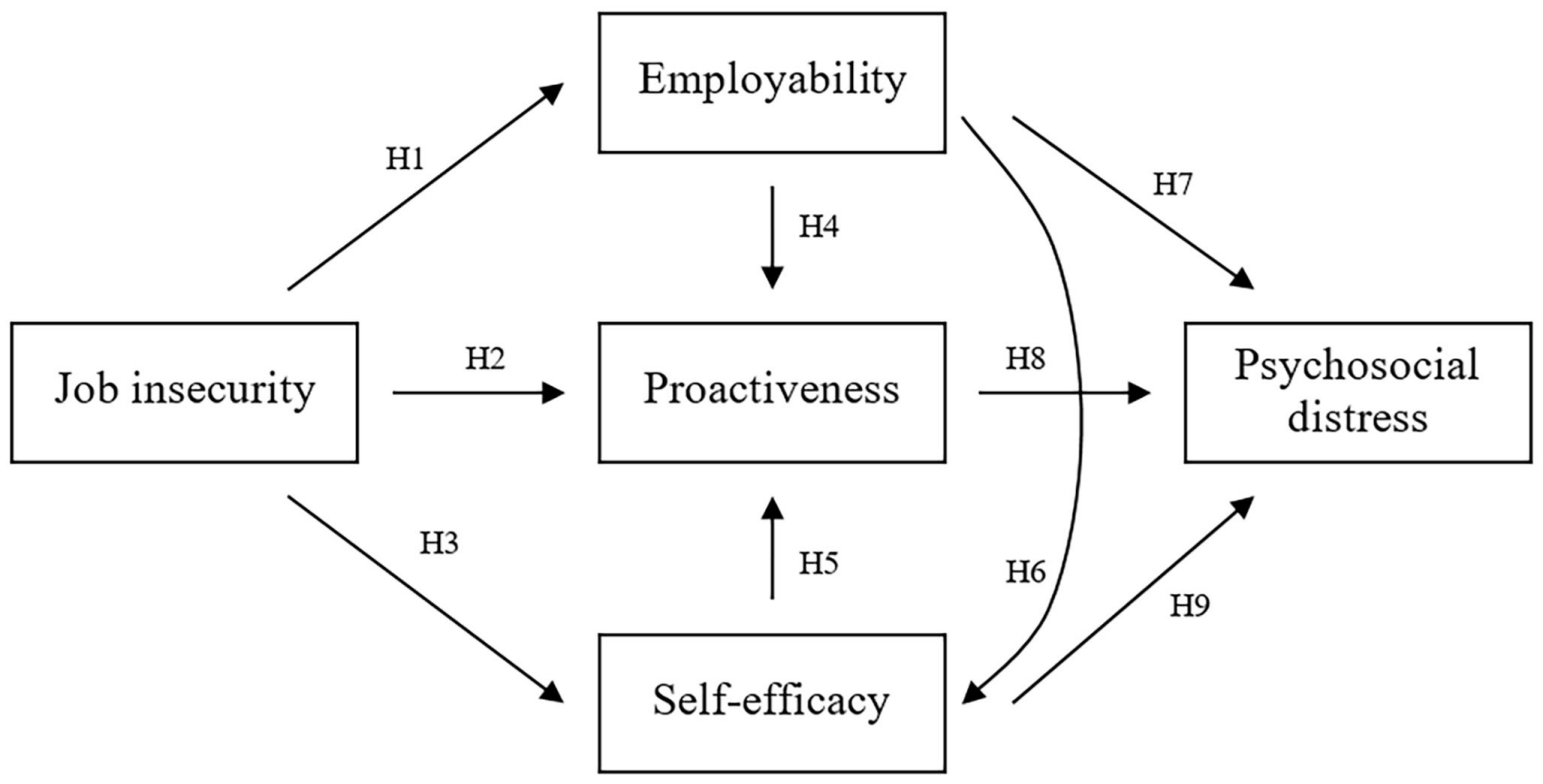
Proposed Theoretical Model

According to the theoretical model presented in Figure 2, the following hypotheses are proposed:

- We expected job insecurity to positively affect employability (H1), and to negatively affect proactiveness (H2) and self-efficacy (H3);
- We expected employability to positively affect proactiveness (H4), and self-efficacy to negatively affect proactiveness (H5) (considering that high scores in self-efficacy indicate low self-efficacy);
- We expected employability to negatively affect self-efficacy (H6) and to positively affect psychosocial distress (H7) (considering that high scores in self-efficacy indicate low self-efficacy and high scores in psychological distress indicate high psychological discomfort);
- We expected proactiveness to positively affect psychosocial distress (H8) and self-efficacy to positively affect psychosocial distress (H9) (considering that high scores in psychological distress indicate high psychological discomfort and high scores in self-efficacy indicate low self-efficacy).

## Method

### Participants and Procedure

Two-hundred and twenty-two jobless, employed and currently unemployed participants, living in Sicily (South Italy), were examined by qualified researchers. Participants were invited to adhere on a voluntary basis, and they were informed about the aims of the study. All participants were asked to complete a self-report questionnaire, which was administered to 211 workers (95% response rate). They were also asked to indicate their gender and age, their education level, and their current and past job position. Sample characteristics are displayed in Table 1.

**Table 1:** Sample Characteristics (*N* = 211)

Variable	Results	
	*N*	%
Gender		
Male	121	57.3
Female	90	42.7
Age		
< 30	39	18.9
30–39	75	36.4
40–50	42	20.4
> 50	50	24.3
Schooling		
Primary school	11	5.2
Secondary school	38	18.0
High school	130	61.6
Graduate	32	15.2
Work condition		
Jobless	101	47.9
Employed	88	41.7
Currently unemployed	22	10.4
Job position in progress or in the past		
Manager	14	6.7
Employee	71	33.6
Workman	67	31.7
Consultant	34	16.2
Unresponsive	25	11.8

### Measures

This study was conducted using a set of questionnaires. The instruments used were the following:

#### The Job Insecurity Scale (JIS)

The JIS (Sverke et al., 2004, Italian version) was used to evaluate the perception of insecurity. It is composed of a five-item scale. The response scale was on a 5-point Likert scale (1 = *strongly disagree*; 5 = *strongly agree*). High scores indicate high job security. Cronbach’s alpha was .85.

#### The Employability Perceived Scale (EPS)

The EPS (Lo Presti & Nonnis, 2008) was used to assess the individual perception about the probability of getting a new job. The tool is based on a five-item scale to which one responds on a 5-point Likert scale (1 = *no probability*; 5 = *100% probability*). Cronbach’s alpha was .90.

#### The Career Transitions Inventory (CTI) for Proactivity (PROACT) and Self-efficacy (SE)

The PROACT and the SE were measured using two CTI subscales (Heppner, Multon, & Johnston, 1994; Lo Presti, 2008). These multidimensional scales were administered to evaluate the perception of psychological resources in career transition workers. The PROACT was assessed by 13 items to which one responds on a 6-point Likert Scale. Cronbach’s alpha was .85. The SE is a tool based on 10 items evaluating on a 6-point Likert Scale the degree of perceived self-efficacy in successfully completing a change in one’s work. High scores indicate low self-efficacy. Cronbach’s alpha was .68.

#### The General Health Questionnaire (GHQ-12)

The GHQ-12 (Goldberg, 1979; Piccinelli, Bisoffi, Bon, Cunico, & Tansella, 1993) is designed to measure the level of individual distress, based on 12 items on a 6-point Likert Scale (1 = *less than usual*; 6 = *more than usual*). High scores indicate high psychosocial discomfort. Cronbach’s alpha was .89.

#### Socio-Demographic Variables

In the last part of the questionnaire, participants provided information about the socio-demographic characteristics, such as gender, age, school grade, occupational history (as years of service), working conditions, and job position, that might be associated with proactiveness, psychological distress and job insecurity.

### Data Analyses

Data were analyzed with the software SPSS (Version 22.0) for Mac. Descriptive analyses were performed using frequencies (percentages). Test scores were reported as mean and standard deviation. Both *t*-tests and one-way analysis of variance (ANOVA) were performed to determine any statistically significant differences between the groups’ means.

Robust maximum likelihood structural equation modelling (SEM) was used to test the hypothesized model. The SEM analyses were performed with EQS Structural Equation Program (Version 6.1; Bentler, 2006). The goodness of fit of the proposed model to the empirical data was evaluated using the Satorra-Bentler scaled chi-square (SBS χ^2^) goodness-of-fit test. As chi-square is sensitive to sample size, we followed Bollen’s (1989) recommendation to use multiple fit indices for interpreting the fit of the model with data. In addition to the chi-square statistic, we examined the chi-square to degrees of freedom (χ^2^/*df*), root-mean-square error of approximation (RMSEA), non-normed fit index (NNFI), and comparative fit index (CFI). Acceptable model fit typically is inferred when RMSEA is .08 or lower, both CFI and NNFI is above .90, and the ratio of chi square to degree of freedom is above 1 and less than 3 (Brown, 2006; Hu & Bentler, 1999).

## Results

### Descriptive Analysis

The results showed statistically significant differences in employability (EPS), proactiveness (PROACT) and job insecurity (JIS) by both participants’ gender and age. That is, different mean scores on the aforementioned measures were found in the comparison between male and female participants, and among the subgroups originated by sample’s age (≤ 30 years; 30–39 years; 40–49 years; ≥ 50).

Table 2 displays statistically significant scores obtained from questionnaires. Job insecurity (JIS) shows statistical differences related to gender (*M* = 3.58 ± 0.302): women show higher scores than their male colleagues (*t* = 3.05; *p* = .003).

**Table 2:** Scores Obtained From Scales

Variable	EPS	PROACT	SE	GHQ-12	JIS
Gender					
Male	1.85 ± 0.94	3.55 ± 0.91	3.01 ± 0.75	2.19 ± 0.67	4.13 ± 3.14
Female	1.65 ± 0.72	3.44 ± 0.87	3.11 ± 0.72	2.38 ± 0.67^a^	2.84 ± 2.71^a^
Age					
≤ 30	2.03 ± 0.71^a^	3.72 ± 0.55^a^	3.08 ± 0.57	2.28 ± 0.56	3.16 ± 2.90
30–39	1.96 ± 0.81^a^	3.78 ± 0.76^a^	3.13 ± 0.70	2.23 ± 0.69	3.39 ± 2.83
40–49	1.69 ± 0.96	3.39 ± 1.01^a^	2.83 ± 0.64	2.21 ± 0.56	3.50 ± 3.20
≥ 50	1.27 ± 0.52^a^	2.86 ± 0.91^a^	23.11 ± 0.91	2.38 ± 0.82	4.53 ± 3.26

*Note*. EPS = Employability Perceived Scale; PROACT = Proactivity; SE = Self-efficacy; GHQ = General Health Questionnaire; JIS = Job Insecurity Scale.

^a^Statistically significant difference.

The scores regarding employability—EPS were significantly lower. As age increased, a progressive reduction of the scores is observed in all five items. ANOVA test shows statistically significant reductions: in age bands > 30 years as to < 50.

Regarding the perception of psychological resources available to the individual who is experiencing a career transition (CTI): PROACT useful for measuring the motivation of individuals in addressing a work transition receives high scores (*M* = 5.92 ± 3.50): as age increased, a progressive reduction of the scores was observed also in this case. Even the scores attributed to the feeling of SE in successfully completing a work change were undoubtedly positive (in this case, low scores corresponded to more positive judgments; *M* = 3.05 ± 7.34).

The measurement of mental and physical discomfort levels (GHQ-12) experienced in recent weeks receives high average scores (3.58 ± 3.02), reflecting high levels of individual malaise. Results reveal statistically significant differences in favor of females (*t* = −2.27, *p* = .024*).*

### Structural Model Testing

We evaluated the structural model shown in Figure 2 and the significance of the hypothesized paths. The proposed theoretical model exhibited good fit with the data. Although the chi-square statistics were significant, SBS χ^2^ (765) = 953.56, *p* < .05, the ratio of chi-square to the degrees of freedom was less than 3, χ^2^/*df* = 1.25, indicating a good level of fit of the structural model with the data. The other fit indices also indicated good fit between the proposed model and the observed data (RMSEA = .04, confidence interval = .31–.47; NNFI = .93; CFI = .94).

**Figure 3:**
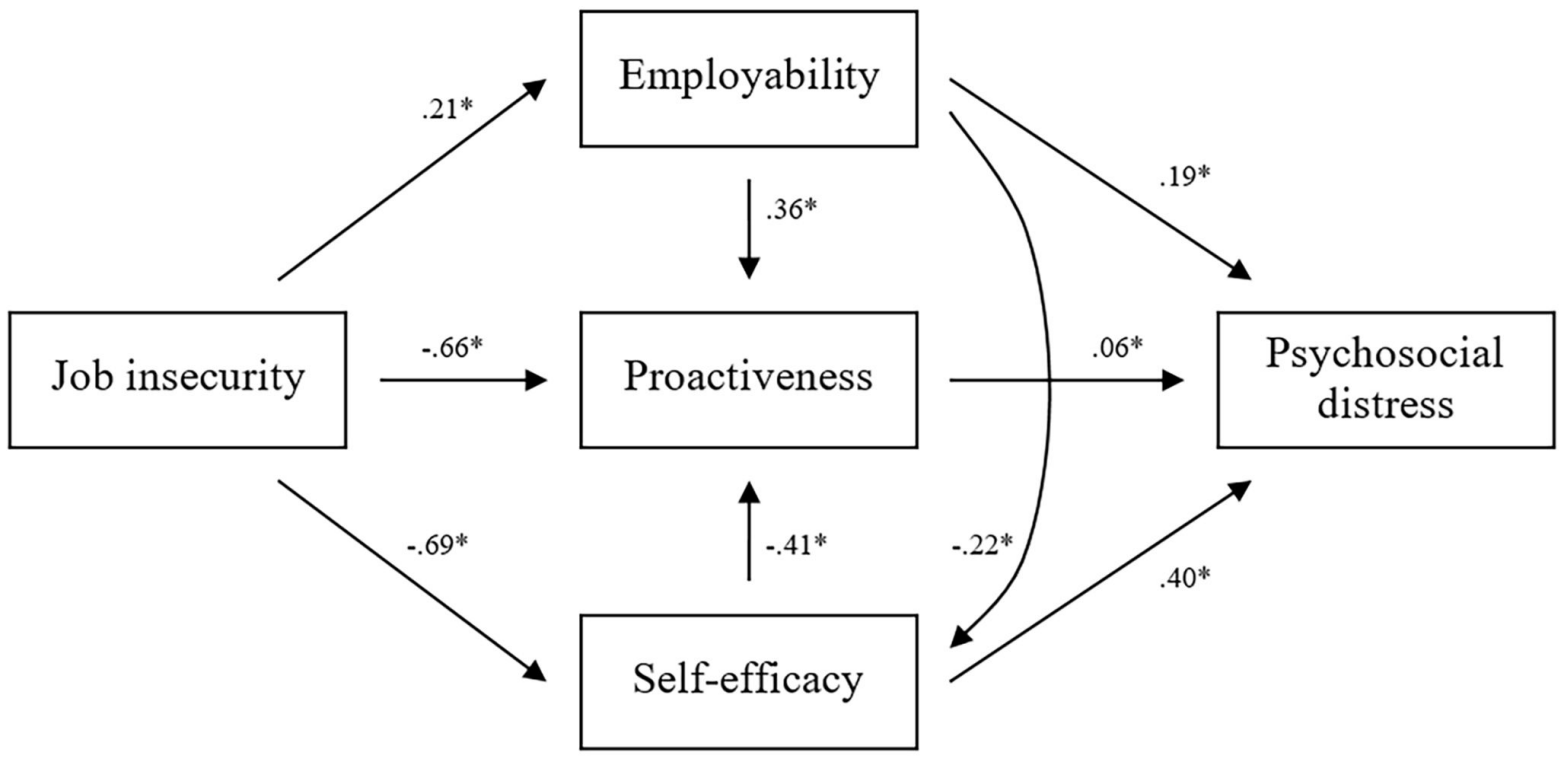
Structural Equation Modelling Results With Standardized Path Estimates *p < .05.

All standardized paths’ coefficients in the model were statistically significant (*p* < .05) and in the expected direction. Specifically, the path running from job insecurity to employability was statistically significant (β = 0.21, *p* < .05), offering support for Hypothesis H1; the path running from job insecurity to proactiveness was statistically significant (β = −0.66, *p* < .05), offering support for Hypothesis H2; the path running from job insecurity to self-efficacy was statistically significant (β = −0.69, *p* < .05), offering support for Hypothesis H3; the path running from employability to proactiveness was statistically significant (β = 0.36, *p* < .05), offering support for Hypothesis H4; the path running from self-efficacy to proactiveness was statistically significant (β = −0.41, *p* < .05), offering support for Hypothesis H5; the path running from employability to self-efficacy was statistically significant (β = −0.22, *p* < .05), offering support for Hypothesis H6; the path running from employability to psychosocial distress was statistically significant (β = 0.19, *p* < .05), offering support for Hypothesis H7; the path running from proactiveness to psychosocial distress was statistically significant (β = 0.06, *p* < .05), offering support for Hypothesis H8; the path running from self-efficacy to psychosocial distress was statistically significant (β = 0.40, *p* < .05), offering support for Hypothesis H9. Thus, all our hypotheses were supported. The SEM results with standardized path coefficients estimates are shown in Figure 3.

## Discussion

The post-modern era is characterized by profound changes in meaning individuals and groups give to work experience. Furthermore, career transitions are becoming more frequent and more challenging in the face of uncertain employment prospects. Employability is thus configured as any worker’s personal characteristics, since it focuses, on the one hand, on the possession of updated professional skills, on the other hand on adaptive skills which make workers capable of increasing their chances of finding employment (Fugate et al., 2004).

Within this framework, this study is aimed to assess the effect of employability and two personal resources (pro-activity and self-efficacy) on the relationship between job insecurity and psycho-social distress (Ramaci, Bellini, Presti, & Santisi, 2019; Ramaci, Pellerone, Ledda, & Rapisarda, 2017).

Our results showed that employability, pro-activeness, psychological distress and job insecurity significantly differed by participants’ gender and age. All the paths running hypothesized in our proposed theoretical model were verified, showing the role of the use of strategies focused on finding employment. Particularly: (1) high levels of employability are positively related to a neat reduction of the time taken to find a job and a better chance of keeping it (Nauta, Vianen, van der Heijden, van Dam, & Willemsen, 2009); (2) the relationship between employability and job placement is mediated by the use of strategies focused on seeking employment (De Battisti, Gilardi, Guglielmetti, & Siletti, 2016); (3) psychological distress appears to be linked to employability, showing a significant relationship with the adoption of non-rational job search strategies (Ingusci, Manuti, & Callea, 2016).

Therefore, the results support all our hypotheses; the negative effects of job insecurity were confirmed, and clear empirical evidence emerged about the positive effects of a versatile career orientation. Although with differentiated effects, these variables positively influenced the perception of self-efficacy necessary to manage their employment situation, with final consequences on the psycho-physical well-being.

A large part of employability research shows the presence of approaches focused on cognitive aspects and perceptions of employability, rather than dispositional factors. Today employability is considered a strategic factor to positively address the changes taking place in the labor market, and introduces a new cultural paradigm around which to rethink actions and interventions to support employment. At the same time, research shows (Berntson et al., 2010; Girardi et al., 2015) that the perception of having a good chance to find a job is associated with a greater sense of job security and greater proactivity.

The discomfort resulting from this condition has clarified how much the labor market and the perception of the worker have changed, requiring new skills and abilities: one should be increasingly flexible and be able to adapt to the various conditions that appear and disappear quickly (Nauta et al., 2009). Employability will help people cope with the transitions and challenges of work in an evolving world of work.

This study contributed to the literature highlighting knowledge on the importance of the emotional dimension in understanding the impact of job insecurity. More specifically, the empirical evidence on the role that personal resources play (Proactive and Self Efficacy) in mitigating the impact of job insecurity on psychological distress makes more sense if it is also reflected in the sampling procedures adopted in research. Usually other research has often used convenience samples; in our case, the sample was selected within the employment offices and with the collaboration of trade union organizations. This does not eliminate the limitations that research presents, but rather offers opportunities for improvement in the future. The results highlight several implications for the development of effective management strategies and practices for the management of problems relating to job insecurity.

### Limitations of Research

Overall, this study found support for the research hypotheses; however, some limitations need to be mentioned. Due to the participants’ characteristics and the small sample size, generalization of our results should be done with caution. Further, all participants were from South of Italy, representing another limit to our findings generalizations. Indeed, Italian southern regions are characterized by a higher level of unemployment and/or unstable jobs compared to northern ones. From this perspective, responses on questionnaires may be influenced by the specific socio-economic context. A heterogeneous sample according to the region of origin may reduce this possible bias. Hence, although consistent with previous findings, the same conclusions may not be warranted in different samples. Another limitation concerns the choice to take into account only one of the subdomains of PsyCap (i.e., self-efficacy), ignoring whether also the other components could mediate the relationship between job insecurity and psychosocial distress. Based on literature (Cai et al., 2015; De Cuyper et al., 2012; Kim et al., 2015; Lo Presti et al., 2018) all the four components of PsyCap are relevant to face issues related to job insecurity. After that, a methodological limitation is related to the lack of investigation of the direct relationship in the hypothesized model. That is, we did not examine how job insecurity affected psychological distress, not allowing to discriminate the direct effect from the indirect ones.

### Conclusions and Implications

Studies on the effects of job insecurity on mental and physical well-being as well as on professional life quality are nowadays well known in literature. This probably also derives from the fact that *career* and *work* are no longer intended as a way of ensuring sustenance but, rather, as contexts endowed with *meaningfulness* (Steger, Dik, & Duffy, 2012; Šverko & Vizek-Vidović, 1995).

The interest in the idea of *meaningful work* has been increased remarkably over the last two decades. Most managerial-based research focused on studying ways of *supplying* and *managing* the *meaningfulness*, for instance through leadership or organizational culture. Most of these researches, however, turn out to be scarce and fragmentary, a large variety of sources of meaningful job being available which, however, do not handle their reciprocal relationships (Faraci, Lock, & Wheeler, 2013; Lips-Wiersma & Morris, 2009).

As Steger et al. (2012) point out, one of the reasons why a *meaningful job* becomes significant is its coherent association with the benefits for workers and organizations. It is well known today that the fundamental dilemmas of managers’ and workers’ lives revolve around the meaningfulness of work (Jackall, 1988; Magnano et al., 2019), whose consequences on psycho-physical well-being are intense and deep (Lips-Wiersma & Morris, 2009). A meaningless job is, indeed, often linked to higher levels of apathy and detachment from it (May, Gilson, & Harter, 2004). *Meaningfulness*, then, refers to the degree whereby life has emotional value and compared requests are perceived as worth of one’s investment and energy commitment (Korotkov, 1998).

From a practical perspective, it seems clear that all of these assumptions take on a remarkable role. In career counseling, the ability to instill *hope* in *finding a job* increases the chances of helping clients to cope with the uncertainties of the future (López, Peón, & Vázquez Ordás, 2004). Results may have impact and great consequences also in terms of flexicurity, carried out by national governments as well as clear outlook/insight as far as the intermediation role and connected practices performed by Labour Agencies.

In conclusion, the present research potentially offers ideas for managerial policies. In such way, focusing on three key-dimensions (i.e., employability, job insecurity and personal resources) becomes an alternative and, likely, more fruitful reading key which can help to enter and return into the labor market, to ensure continuity and careers, as well as to give significance to one’s work identity.

Our findings can be considered as a relevant contribute addressing nowadays work-related conditions. From this point of view, employability, self-efficacy, and proactiveness can be seen as suitable protective factors and, for this reason, they should be promoted and encouraged.
